# Anthropometric measures do not explain the 2D:4D ratio sexual dimorphism in 7‐year‐old children

**DOI:** 10.1002/ajhb.23776

**Published:** 2022-06-25

**Authors:** Zebulohn Jägetoft, Maria Unenge Hallerbäck, Maria Julin, Carl‐Gustaf Bornehag, Sverre Wikström

**Affiliations:** ^1^ Centre for Research and Education County Council of Värmland Karlstad Sweden; ^2^ Department of Health Sciences Karlstad University Karlstad Sweden; ^3^ School of Medical Science Örebro University Örebro Sweden; ^4^ Västerås Hospital County Council of Västmanland Västerås Sweden; ^5^ Department of Preventive Medicine Ichan School of Medicine at Mount Sinai New York New York USA

## Abstract

**Objectives:**

Digit ratio (2D:4D) might reflect prenatal testosterone exposure and has been used as a putative marker for androgen related outcomes. However, such associations might be inflicted by confounders. Application of 2D:4D in epidemiological research motivate identification of biological background determinants.

We examined sex, anthropometric measures, and maternal factors as determinants of 2D:4D in Swedish 7‐year‐old children.

**Methods:**

The study was embedded in the Swedish Environmental, Longitudinal, Mother and Child, Asthma and Allergy (SELMA) pregnancy cohort. A total of 870 pre‐pubertal children, median 7.5 years of age, were studied.

A single assessor performed digit measurements from scanned photocopies using computer software. Child anthropometric measurements investigated were hand size, birthweight, recumbent birth length, standing height, weight, BMI, body fat percentage, and waist/hip circumference. Maternal factors included age, pregnancy length, parity, and education.

**Results:**

We found a significant sexual dimorphism regarding digit lengths and 2D:4D, boys on average presenting a lower 2D:4D than girls also after adjustment for summed finger lengths and body fatness. In crude analyses, maternal age correlated with 2D:4D across the whole population and in females but not in adjusted models. No other study variables were associated with 2D:4D.

**Conclusion:**

Digit ratio showed sexual dimorphism at the age of seven and seems to represent a true sex difference rather than an artifact and bias from hand size, body size or body fat content. Among the rest of our investigated variables, we found no determinants constituting important confounders in future research on 2D:4D ratio.

## 1 INTRODUCTION

In utero hormones effect on fetal development have been extensively examined and associated with different future outcomes, like adolescence behavioral characteristics (Day et al., 2020). Testosterone is considered one of the most important prenatal hormones through its participation in fetal differentiation of the brain (Lombardo et al., 2012) and reproductive tissues (Wolf, 2002).

The estimation of fetal hormonal milieu is complex. Direct procedures, like amniocentesis (van de Beek et al., 2004), can only be acquired around the second trimester and onward. As well, amniocentesis could be afflicted by potential fetal risk (Mujezinovic & Alfirevic, 2007). Although less invasive procedures, like analyses of circulating hormones in maternal blood, hair or saliva, may reflect fetal hormonal exposure, they are potentially influenced by hormone production from other sources (e.g., placenta, the fetus, or the mother herself) and inconsistency across methods (O'Connor & Barrett, 2014). Therefore, any easily gained proxy marker for fetal hormonal environment may be appealing especially in large‐scale epidemiological research.

Besides effect on neurodevelopment and reproductive tissues, in utero testosterone is also thought to regulate the expression of HOX‐genes (Daftary & Taylor, 2006), partly responsible for fetal digit length development (Kondo et al., 1997). The ratio between the index finger and ring finger lengths (2D:4D) has therefore been proposed to serve as a proxy marker for in utero testosterone exposure (Manning et al., 1998). This hypothesis have been verified on androgen receptor (AR) knock out mice, where inactivation of AR lead to accelerated growth of the 4th digit, consequently resulting in lower digit ratio (Zheng & Cohn, 2011). Human research on fetal hormones obtained from amniocenteses supports a relationship between low right hand 2D:4D and high fetal testosterone relative to fetal estradiol (Lutchmaya et al., 2004). Similarly, other studies have found altered digit ratio in people with androgen‐related disorders, like congenital adrenal hyperplasia (CAH, Brown et al., 2002) and Klinefelter's syndrome (Manning et al., 2013). Correspondingly, studies on healthy populations have found a sexual dimorphism in 2D:4D, where males tend to present a lower 2D:4D ratio than females, proposedly due to males' higher exposure to prenatal testosterone (Jeevanandam & Muthu, 2016; Manning et al., 1998). Digit ratio is considered to be established prenatally (Galis et al., 2010; Manning et al., 1998), but data regarding sex difference of the finger ratio during childhood is less robust as compared with adult findings (Arbuckle et al., 2018; Barrett et al., 2020; Manning & Fink, 2018; Rizwan et al., 2007).

Thus, as 2D:4D might provide an insight in fetal milieu, publications have focused on 2D:4D as a predictor for diseases of fetal origin. For example, association between low 2D:4D and presence of neuro developmental disorders (NDDs) (Al‐Zaid et al., 2015; De et al., 2007; Manning et al., 2001), impairments thought to partly arise from androgenic effect on fetal neurodevelopment (Kelemenova & Ostatnikova, 2008). Similarly, 2D:4D have also been utilized in investigations regarding correlation with other human traits like depression (Bailey & Hurd, 2005) and cardiovascular risk factors (Oyeyemi et al., 2014). Still, the utilization of digit ratio as marker for testosterone's prenatal effects is questioned and the scientific basis and use are not agreed upon (Leslie, 2019). For instance, findings from a recent study rejected the association between 2D:4D, sex and other hormone‐sensitive measures altogether (Barrett et al., 2020). Investigations on digit ratio in relation to health‐ and behavioral outcomes may be confounded by co‐varying determinants. As an illustration, publications have proposed that the 2D:4D ratio might be influenced by sex differences in absolute finger lengths (Kratochvíl & Flegr, 2009) or digit adiposity (Wallen, 2009). Furthermore, 2D:4D associations to anthropometric measurements such as weight, height, and BMI have been inconsistent (Almasry et al., 2011; Barut et al., 2008; Fink et al., 2003; Klimek et al., 2014; Muller et al., 2013).

Consequently, in order to optimize utilization of digit ratio in a wider range of research questions, biological background determinants of 2D:4D, constituting potential confounders in association with, for example, behavioral outcomes need to be identified. We here examined sex, anthropometric measures and maternal factors as determinants of 2D:4D in a large cohort of Swedish seven‐year‐old pre pubertal children.

## 2 METHOD

### 2.1 Study overview and population

This study was embedded in the ongoing Swedish Environmental Longitudinal, Mother and Child, Asthma and Allergy Study (SELMA) pregnancy cohort. All pregnant women registered at an antenatal care center during September 2007 to March 2010 in Värmland, Sweden were invited. Women beyond gestational week 22 and those who did not understand written Swedish were excluded. A total of 6658 mothers‐to‐be were assigned to take part in SELMA of which 2582 (39%) chose to participate after written consent. During 2015 until 2017, a random sample of 1500 children at 7 years of age from SELMA was invited to a follow up examination. Initial information was provided in written form as well as through educational videos addressing the children. Children were then included after parental consent. During the exam, participants had their hands photocopied and underwent a variety of body exams and blood sampling at the local health care center. The body exams were performed by trained nurses. Questionnaires were filled in by the parents. A total of 1003 children participated and were thus eligible for inclusion in this study.

The study was approved by the regional ethics committee in Uppsala, Sweden (2007‐05‐02, Dnr: 2007/062 and 2015‐06‐10, DNR: 2015/177) and performed in accordance with Declaration of Helsinki.

### 2.2 Digit measurement

All children were instructed to place both hands palmar side down on a flatbed photocopy scanner (Kyocera TASKalfa 306ci, Kyoto, Japan) without rings, watches, and so forth. A research nurse monitored the process to make sure all hands were equally and correctly placed on the glass bed. Each photocopy also included the participant's coded study‐id and a control ruler. Photocopies were scanned to digital format (.pdf) and contrast was optimized in order to facilitate digital measurement. Images which were indiscernible even after optimization were excluded. Digit lengths were then measured by a single assessor (Zebulohn Jägetoft) on the right hand, between the midpoint of most proximal palmar crease and the tip of the digit, using Adobe Photoshop (Adobe Inc., California, USA), with an accuracy of one tenth of a millimeter. After first evaluation and measurement, photocopies with significant deviated/flexed fingers or vague palmar crease/fingertip underwent a second opinion by another trained investigator (Sverre Wikström) before either remeasured by Zebulohn Jägetoft, or excluded. Also, in order to avoid dependent data due to indistinguishable genome, all twin pairs and triplets were excluded from the analysis. Finally, 2D:4D was calculated as a ratio between the length of the index and ring finger.

For quality control of digit measurements, 100 participants underwent a second assessment by a third trained assessor (Maria Julin), blinded to the first round of measurements. An inter‐rater correlation coefficient (ICC) was then calculated to evaluate consistency. The ICC was *r* = .994 (*p* < .001) for 2D and *r* = .995 (*p* < .001) for 4D, indicating high reliability.

### 2.3 Anthropometric characteristics

The anthropometric measurements used in the present study were birthweight, recumbent length at birth, and by the age of seven the current standing height, body weight, body mass index (BMI), body fat percentage and waist and hip circumferences. Standing height was measured with a 0.1 cm accuracy using a Seca 217 stadiometer (Seca GmbH, Hamburg, Germany). Waist circumference was measured, midway between the lower rib margin and lateral point of the iliac crest, in a standing position at the end of a normal exhalation using a Seca 201 measuring tape. Hip circumference was measured using the same measuring tape at the widest part of the hip. Body fat percentage was measured using bio‐impedance analysis (BIA) on a Tanita BC‐418 device, (Tanita Corp. Tokyo, Japan), also measuring body weight (with a 0.1 kg accuracy). Measurements were taken without clothes, before breakfast and with an empty bladder. Maternal determinants included age, pregnancy length (days according to ultrasonography), parity and education level (dichotomized as university or less) which was provided from study questionaries' and/or the national Swedish Medical Birth Register.

### 2.4 Statistics

Statistical analyses for sex differences in continuous variables were performed using unpaired *t*‐test or Mann–Whitney *U*‐test, based on the distribution of the respectively variables. The proportions of girls and boys respectively with 2D:4D > 1 were calculated and compared with Fisher's exact test. Analyses for maternal categorical variables and 2D:4D were performed with unpaired *t*‐test. For correlations between 2D:4D and the anthropometric measurements, Spearman's rank correlation analysis was utilized.

In a multiple linear regression model, sex was thereafter investigated as predictor of 2D:4D ratio, adjusting for the sum of 2D + 4D lengths. Finally, variables of potential importance due to crude (i.e., bi‐variate) association with 2D:4D were chosen as co‐variates in a multiple regression model, a priori already including sex, sum of 2D + 4D lengths (i.e., adjustment for hand size) and age in months (for comparison with previous research) (Barrett et al., 2020).

Statistical analyses were achieved using IBM SPSS Statistic version 26 (IBM Corporation, New York, USA). *p*‐values <.05 were considered significant.

## 3 RESULTS

**FIGURE 1:**
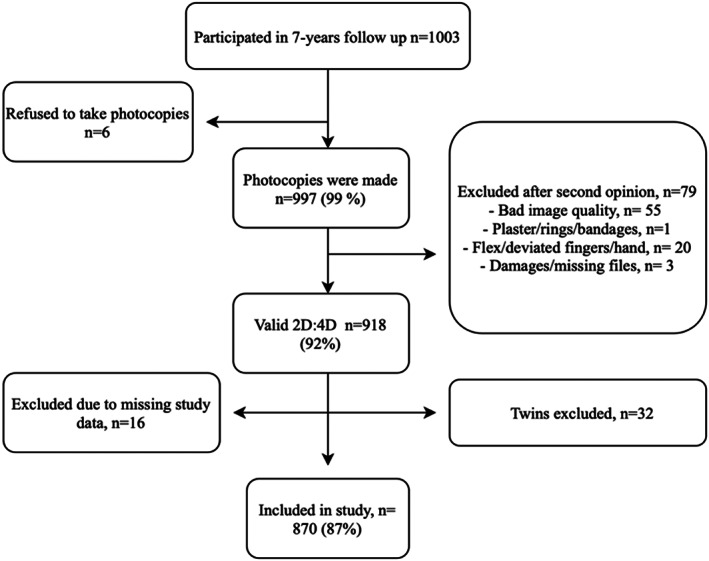
Flowchart of study participants and exclusion process

A total of *n* = 1003 children participated in the follow up exam and all were verified to be before puberty onset. Median (IQR) age by examination was 7.5 (7.35–7.64) in both boys and girls. Six children refused to take photocopies of their hands; thus, a total of *n* = 997 photocopies were performed. Due to poor image quality, deviated finger/hand or damaged files, *n* = 918 digit lengths were measured. After final data merging with the overall SELMA study database variables and exclusion of twins, a total of *n* = 870 children were successfully included in final analyses, as presented in Figure 1.

The associations between anthropometrics and sex are presented in Table 1. We found a sex difference regarding birth weight, birth length as well as body fat percentage, standing height and hip circumference at 7 years of age. We also found significant sex differences regarding lengths of 2nd digit and 4th digit (both longer in males), as well as 2D:4D ratio, (lower in males, Table 1). The proportion of girls with 2D:4D > 1 was 5.5% as compared with 2.7% in boys (*p* = .05).

**TABLE 1:** Association between anthropometric characteristics of the study sample by sex

	Females (*n* = 431)	Males (*n* = 439)	*p*‐value*
Maternal anthropometrics			
Age at birth (y)	31 (4.9)	31 (4.4)	.74
Pregnancy length (d)	281 (274–287)	281 (273–287)	.85
University education (%)	261 (61.7%)	279 (65.3%)	.27
Multiparous (%)	238 (55.6%)	230 (52.5%)	.36
Child anthropometrics			
Birth weight (g)	3530 (3205–3879)	3700 (3370–4115)	<.01
Birth length (cm)	51 (49.5–52)	52 (50–53)	<.01
Body weight (kg)	25.7 (23–29.1)	26 (23.8–29)	.09
Standing height (cm)	127.6 (5.6)	129.2 (5.6)	<.01
Body mass index (BMI)	15.6 (14.6–17.2)	15.6 (14.7–16.8)	.78
Body fat percentage (%)	22.7 (20.8–25.4)	20.2 (18.6–22.6)	<.01
Waist circ. (cm)	56.4 (53.5–60.5)	56.6 (54–59.8)	.24
Hip circ. (cm)	66 (63–69.5)	65 (62–68.3)	.01
2nd digit (cm)	5.325 (0.312)	5.379 (0.337)	.02
4th digit (cm)	5.593 (0.323)	5.682 (0.346)	<.01
2D:4D	0.953 (0.031)	0.947 (0.030)	.01

*Note*: Number presented as medians (IQR) for non‐normally distributed data and mean (SD) for normally distributed data. *p*‐values for difference between groups.

Abbreviations: 2D, second digit; 4D, fourth digit; 2D:4D, second‐to‐fourth digit ratio; *n*, numbers; d, days; y, years; cm, centimeters; %, percentage; g, grams.

* *p*‐value for difference between females and males. From Mann–Whitney *U*‐test for non‐normally distributed continuous variables, from unpaired *t*‐test for normally distributed continuous variables and from Pearson's chi‐square for categorical variables.

As presented in Table 2, we identified a linear correlation between body fat percentage and 2D:4D, and between maternal age at birth and 2D:4D across the whole population. However, in sex stratified analyses no correlation between body fat percentage and 2D:4D remained and maternal age at birth was only correlated with female 2D:4D. No other significant associations between 2D:4D and the investigated variables were observed (Tables 2 and 3).

**TABLE 2:** Correlation analysis between right hand 2D:4D and anthropometric measurements as well as maternal variables

Study variables		2D:4D—All, *n* = 870	2D:4D—Females, *n* = 431	2D:4D—Males, *n* = 439
Sum 2D + 4D	*r*_*s*_	.012	.000	.042
	*p*‐value	.726	.994	.378
Birth weight	*r*_*s*_	−.007	.016	−.014
	*p*‐value	.835	.743	.765
Birth length	*r*_*s*_	−.026	.016	−.043
	*p*‐value	.448	.737	.371
Body weight	*r*_*s*_	.001	−.021	.032
	*p*‐value	.971	.660	.510
Standing height	*r*_*s*_	.026	.018	.052
	*p*‐value	.453	.706	.281
Body mass index (BMI)	*r*_*s*_	−.012	−.034	.006
	*p*‐value	.756	.522	.909
Body fat percentage	*r*_*s*_	.072	.052	.067
	*p*‐value	.035	.287	.162
Waist circumference	*r*_*s*_	.000	−.012	.015
	*p*‐value	.999	.802	.751
Hip circumference	*r*_*s*_	.025	−.019	.062
	*p*‐value	.461	.697	.197
Pregnancy length	*r*_*s*_	−.006	.025	−.032
	*p*‐value	.862	.606	.507
Maternal age	*r*_*s*_	−.089	−.115	−.022
	*p*‐value	.044	.018	.653

*Note*: Numbers presented as rank correlation coefficients (*r*_*s*_). Correlation using Spearman's rank correlation analysis.

Abbreviation: 2D, second digit; 4D, fourth digit; 2D:4D, second‐to‐fourth digit ratio.

**TABLE 3:** 2D:4D ratios separated by mother's parity and education

	Primiparous	Multiparous	*p*‐value*
2D:4D			
Total (*n* = 866)	0.952 (0.029)	0.948 (0.032)	.089
Female (*n* = 428)	0.955 (0.029)	0.951 (0.032)	.270
Male (*n* = 438)	0.949 (0.030)	0.945 (0.031)	.153

	Maternal high school education or lower	Maternal university education	*p*‐value*
2D:4D			
Total (*n* = 850)	0.950 (0.030)	0.949 (0.031)	.657
Female (*n* = 423)	0.953 (0.030)	0.952 (0.031)	.838
Male (*n* = 427)	0.947 (0.031)	0.947 (0.031)	.769

*Note*: Number presented as mean (SD). *p*‐values for difference between groups.

Abbreviation: 2D:4D, second‐to‐fourth digit ratio.

* From unpaired *t*‐test.

In multiple linear regression, there was an association between sex and 2D:4D, also when adjusting for the sum of 2D +4D lengths (*β* = −.006 and standardized regression coefficient = −.095; *p* = .005 for 2D:4D in boys as compared with girls). In the final regression model with co‐variates based on bi‐variate associations and theoretical considerations as above, sex was included as predictor along with the sum of 2D + 4D; child age; maternal age and body fat percentage. Here, only sex was identified as significant predictor of 2D:4D (*β* = −.007 and standardized regression coefficient = −.110; *p* = .007 for 2D:4D in boys as compared with girls). The associations between all investigated anthropometric study variables respectively, as well as in relation to 2D:4D ratio and sex, are summarized in Supplementary Table S1.

## 4 DISCUSSION

We investigated determinants of 2D:4D ratio in 870 Swedish pre‐pubertal 7‐year‐old‐children, aiming to identify confounders of importance for future analyses of 2D:4D in relation to health‐ or behavioral outcomes. We found that 2D:4D digit ratio was significantly different between sexes already at the age of seven, with males on average presenting a lower 2D:4D than females, and with a lower proportion of males than females having 2D:4D > 1. This is in line with several previous studies, also reporting a sex difference of 2D:4D already before puberty onset and during adolescence (Körner et al., 2020; Manning et al., 1998; Rizwan et al., 2007; Velez et al., 2017; Wong & Hines, 2016), although this less pronounced in early ages (Arbuckle et al., 2018; Manning & Fink, 2018) as compared with studies on adult subjects. However, there are also studies questioning the typical sex dimorphic pattern in early ages (Hönekopp & Thierfelder, 2009; Saenz & Alexander, 2013). Recently, Barrett et al. (2020) reported on 321 pre‐pubertal children and found no sex difference in 2D:4D after adjusting for age, race, and study center. They nonetheless applied direct measurements of digits with calipers that, as pointed out by the authors, lack the greater quality control opportunity of indirect measurements on a less time sensitive basis (Barrett et al., 2020; McIntyre et al., 2006). It may be speculated that this methodological difference, together with a larger number of examiners (Barret et al. utilized two or three per study site and in total four sites, as compared to a single rater in our study) explain the dispersed measurement outcomes. It may also be noticed that mean participant age also differs, being 4.5 years in the study by Barret et al. and 7.5 years in the present study. In a similar late pre‐pubertal age as in our study, Rizwan et al. (2007) performed 2D:4D investigation on 520 children (mean age 8.3 years as compared with our 7.5 years) using direct measurements and found a significant lower 2D:4D in boys right hand.

The originally suggested biological explanation for the sex dimorphic phenotype of 2D:4D is an androgen‐related pattern of digit lengths (Zheng & Cohn, 2011). Yet, (Kratochvíl and Flegr (2009) suggested that the sex difference in 2D:4D may be confounded by covariation with the absolute difference in digit length between males and females, hence proposing that 2D:4D is a side‐effect of allometric shift in digit ratio with digit lengths, rather than a product of androgenic influence. In our study, both 2D and 4D lengths were significantly different between sexes but there was no correlation between digit ratio and the sum of 2D + 4D lengths. Despite this, but in order to further investigate any importance of overall finger lengths on the association between sex and 2D:4D, we adjusted our analyses for the sum of 2D + 4D with no impact on the identified sex difference.

In another publication on 2D:4D, Wallen stated that 2D:4D ratio difference is more likely an indicator for sex difference in digit adiposity (Wallen, 2009), rather than proxy marker for fetal testosterone. In our analyses we found an expected difference in body fat percentage between girls and boys and a minor correlation between body fat percentage and 2D:4D across the whole study population, but importantly not when stratified by sex. Since girls have in general higher body fat percentage, as well as a higher 2D:4D, the weak positive correlation between body fat percentage and 2D:4D was probably confounded by sex. Likewise, we found no correlation between BMI or waist/hip ratio and 2D:4D, hence further rejecting an association between body size/fatness and 2D:4D. This is in line with a meta‐analysis on 2D:4D which similarly rejected the proposed correlation between 2D:4D and digit adiposity (Hönekopp & Watson, 2010). So far, other studies on the associations between 2D:4D and overall anthropometric measures have been inconsistent. Some have presented a negative correlation between 2D:4D and BMI (Almasry et al., 2011), and some a positive correlation (Fink et al., 2003). In a large scale study with more than 14 000 participants and digits measured from photocopies, Muller et al. (2013) presented results similar to ours, with no correlation between 2D:4D and adult anthropometric measurements. Nevertheless, mean participant age were approximately 54 years old. As of pre‐pubertal and adolescent study populations, a study performed by Klimek et al. investigated digit ratio and anthropometrics in 320 children and youths (3–22 years old; mean 10.8 years) and found an association between 2D:4D, birth length and childhood/adolescence BMI. Although Klimek et al. (2014) used dichotomized 2D:4D (as high vs. low) in the main analyses, which could be considered a limitation, post hoc analyses of 2D:4D as continuous variable still showed that right hand 2D:4D was negatively correlated to body mass and BMI. In the present sample more than twice as large, we did not reproduce this finding.

In our multiple regression model (including both sex and body fat percentage), there was no evidence of association between body fat and 2D:4D (regression coefficient *β* < .001, *p* = .132). Altogether, including all the previous inconsistencies above, and the lack of association with other body measures such as BMI in the present analyses, we consider the weak correlation between body fat percentage and 2D:4D in the overall study sample to be scientifically irrelevant. In summary, we conclude that the sex difference in 2D:4D, as reported among different populations, seems to represent a true sex dimorphism, rather than an artifact and bias from hand size or body fat.

In crude analyses we found a statistically significant negative correlation between maternal age at birth and 2D:4D across the whole population. In stratified analyses this correlation remained significant only for female digit ratio. To our knowledge, no other studies have investigated the correlation between maternal age and 2D:4D. Any biological explanation for such a negative correlation remains unknown. However, a Swedish study showed that amniotic fluid testosterone was negatively correlated with maternal age (Kallak et al., 2017), findings that contradict our results of a lower 2D:4D (putative for higher fetal testosterone) among older mothers. Since our multi‐variate model did not support an association between maternal age and 2D:4D (regression coefficient *β* < .001, *p* = .140) we find it premature to regard mothers' age a confounder in future studies on 2D:4D.

As of pregnancy length, mothers' parity, and education, we did not find any significant association with 2D:4D. To our knowledge, no previous study has investigated these maternal variables as determinants of 2D:4D. Nonetheless, Kallak et al. reported an association between higher prelabor amniotic fluid testosterone and both primiparity as well as increased gestational age in 56 female fetuses (Kallak et al., 2017). Our present investigation may hence provide information for future studies, indicating no need to adjust analyses focusing on 2D:4D ratio for pregnancy length, parity, or education level.

When using digit ratio in research, differences in measurements techniques could result in disperse outcomes across studies. Assessments with radiographic images may be the least vulnerable to soft tissue disturbance but is not so easily acquired in large‐scale epidemiological research. Digit ratio is more often measured either through direct (e.g., calipers) or indirect (e.g., photocopies) methods. In the present study we used indirect computer‐based measurements from scanned photocopies. Although no gold standard has emerged for 2D:4D investigations, previous studies have used indirect methods with comparable, high interclass correlation coefficient and could therefore be considered an adequate method (Myers et al., 2018). Advantages of indirect measurement include the possibilities of quality assessments, remeasurements, and contrast optimization, as applied in present study. Importantly, by using coded photocopies we ensured blinding throughout the whole measuring process. Furthermore, digit measurements were performed by a single trained assessor to ensure most possible consistency across all digit measurements, but we also found a high ICC in a sub sample remeasured by another equally trained assessor. A total of *n* = 79 photocopies (corresponding to 8% of the final sample) were excluded in final analyses after second opinion. Most photocopies (*n* = 55) were excluded due to bad images quality resulting in indiscernible measuring points. Such copying mistakes should be considered in the study design and quality control of future studies including scanned photocopies. Another limitation of the present study is the restriction of measurements to the right hand only. Several previous studies have utilized digit ratio from both hands for evaluation. Yet, previous investigations have suggested a potentially more pronounced correlation between fetal sex hormones and 2D:4D in the right hand, which underlies our decision (Hönekopp & Watson, 2010; Lutchmaya et al., 2004).

Finally, our study population was restricted to a narrow age interval. Although the majority of studies, as already described above, show that 2D:4D is relatively stable regardless of age (Manning & Fink, 2018; Manning et al., 1998, 2004) one study have suggested an increase in 2D:4D with age (Cho & Kim, 2015), potentially reducing the replicability of our results in other populations. Our findings therefore represent boys and girls some years before puberty onset and may be important for future epidemiological studies on 2D:4D in relation to behavioral outcomes.

## 5 CONCLUSION

We found that the 2D: 4D ratio was significantly lower in males than females already at the age of seven and adjusted for overall finger lengths as well as body fat. From our investigations on 2D:4D ratio in relation to various anthropometric measures, we conclude that this difference in 2D:4D, as now reported among different populations, represents a true sex dimorphism, rather than an artifact and bias from hand size or body fatness. As well, 2D:4D is not associated to pregnancy length, parity or maternal education. Despite a weak association in bi‐variate analyses without stratification by sex, from multivariate analyses maternal age seems to lack association with 2D:4D and cannot be regarded a potential confounder for assessment in future research.

## AUTHOR CONTRIBUTIONS

**Zebulohn Jägetoft:** conceptualization, formal analyses, investigation, methodology, visualization, writing—original draft preparation. **Maria Unenge Hallerbäck:** conceptualization, methodology, supervision, validation, writing—review and editing. **Maria Julin:** conceptualization, methodology, writing—review and editing. **Carl‐Gustaf Bornehag:** conceptualization, funding acquisition, supervision, writing—review and editing. **Sverre Wikström:** conceptualization, formal analyses, methodology, supervision, validation, writing—review and editing.

## FUNDING INFORMATION

This research did not receive any specific grant from founding agencies in the public, commercial or not‐for‐profit sectors. The SELMA study was supported by grants from the Swedish Research Council for Environment, Agricultural Sciences and Spatial Planning (Formas) and the County Council of Värmland. Zebulohn Jägetoft was supported by the Region Örebro County ALF‐agreement.

## CONFLICT OF INTEREST

The authors declare no potential conflict of interest.

## Supporting information


**Supplementary Table S1** Summary of associations between all anthropometric study variables respectively (bivariate correlations), as well as in relation to sex and 2D:4D ratio (with adjustments in multiple regression models). *Statistically significance *p* < .05

## Data Availability

Anthropometric data can be made available to researchers upon request (subject to a review of secrecy). Requests for data should be made to the Head of Department of Health Sciences, Karlstad University. However, unique combinations of any data that may make a study participant identifiable, will not be shared.
