# Supplementary material for: Anthropometric measures do not explain the 2D:4D ratio sexual dimorphism in 7‐year‐old children
Source: Am J Hum Biol. 2022 Jun 25;34(9):e23776. doi: 10.1002/ajhb.23776 (PMC9540332; doi:10.1002/ajhb.23776)
Supplement: Supplementary file 1 — Supplementary Table S1 Summary of associations between all anthropometric study variables respectively (bivariate correlations), as well as in relation to sex and 2D:4D ratio (with adjustments in multiple regression models). *Statistically significance p < .05 [file AJHB-34-e23776-s001.docx]

**Supplementary table 1.** Summary of associations between all anthropometric study variables respectively (bivariate correlations), as well as in relation to sex and 2D:4D ratio (with adjustments in multiple regression models). * denotes statistically significance p<0.05.

|  | 2D | 4D | 2D+4D | 2D:4D | Weight | Height | BMI | Waist circ | Hip circ | Body fat% | Sex |
| --- | --- | --- | --- | --- | --- | --- | --- | --- | --- | --- | --- |
| 2D |  | * | * | * | * | * | * | * | * | * | * |
| 4D |  |  | * | * | * | * | * | * | * | * | * |
| 2D+4D |  |  |  |  | * | * | * | * | * | * | * |
| 2D:4D |  |  |  |  |  |  |  |  |  |  | * |
| Weight |  |  |  |  |  | * | * | * | * | * | * |
| Height |  |  |  |  |  |  | * | * | * | * | * |
| BMI |  |  |  |  |  |  |  | * | * | * |  |
| Waist circ |  |  |  |  |  |  |  |  |  | * |  |
| Hip circ |  |  |  |  |  |  |  |  |  | * | * |
| Body fat% |  |  |  |  |  |  |  |  |  |  | * |
| Sex |  |  |  |  |  |  |  |  |  |  |  |

2D, second digit; 4D, fourth digit; 2D:4D, second-to-fourth digit ratio; BMI, body mass index
